# Physiological Responses of an Arctic Crustose Coralline Alga (*Leptophytum foecundum*) to Variations in Salinity

**DOI:** 10.3389/fpls.2020.01272

**Published:** 2020-08-19

**Authors:** Arley F. Muth, Andrew J. Esbaugh, Kenneth H. Dunton

**Affiliations:** Department of Marine Science, University of Texas Marine Science Institute, Port Aransas, TX, United States

**Keywords:** crustose coralline algae (CCA), physiology, salinity, calcification, Arctic

## Abstract

In the Beaufort Sea, Arctic crustose coralline algae (CCA) persist in an environment of high seasonal variability defined by naturally low pH ocean water and high magnitude freshwater pulses in the spring. The effects of salinity on the CCA *Leptophytum foecundum* were observed through a series of laboratory and field experiments in Stefansson Sound, Alaska. We found that salinity (treatments of 10, 20, and 30), independent of pH, affected *L. foecundum* physiology based on measurements of three parameters: photosynthetic yield, pigmentation, and calcium carbonate dissolution. Our experimental results revealed that *L. foecundum* individuals in the 10-salinity treatment exhibited an obvious stress response while those in the 20- and 30-salinity treatments were not significantly different for three parameters. Reciprocal *in situ* transplants and recruitment patterns between areas dominated by CCA and areas where CCA were absent illustrated that inshore locations receiving large pulses of freshwater were not suitable for CCA persistence. Ultimately, spatially and temporally varying salinity regimes levels affected distribution of CCA in the nearshore Arctic. These results have implications for epilithic benthic community structure in subtidal areas near freshwater sources and highlight the importance of salinity in CCA physiology.

## Introduction

Calcification of marine organisms is of broad and current interest in ocean climate change studies. Lower pH levels, driven by carbon dioxide (CO_2_) uptake into ocean water, causes a reduction of calcium carbonate (and other forms e.g., aragonite and calcite) saturation levels leading to decreased calcification rates of species. At high latitudes, aragonite saturation levels are low (~2) when compared to ocean averages (~3.5) or low latitude values (~4; Gangstø et al., 2008; Jiang et al., 2015) due to processes such as freshwater input, rapid seasonal ice melt, upwelling, and relatively high respiration rates from decomposition of organic matter (Mathis et al., 2015). In the Arctic Ocean, natural factors in addition to anthropogenic factors have the potential to decrease aragonite saturation levels below natural variations (**Ω**_Arag_ 3.5–0.8) by 2025 (Mathis et al., 2015).

Decreased rates of productivity, growth, and net calcification have been shown to occur when coralline algal species are exposed to lower than ambient pH conditions for short time periods (~21 days; Büdenbender et al., 2011; Diaz-Pulido et al., 2012; Mccoy, 2013). Crustose coralline algae (CCA) species are often the first species affected by lower pH because they precipitate magnesium calcite, which is about 20% more soluble than aragonite (depending on magnesium content; Morse et al., 2006; Kamenos et al., 2008). CO_2_ vents in Italy decrease pH locally and coralline species disappeared near the vents while turf algal biomass increased 60%, including certain invasive species (Hall-Spencer et al., 2008). Low salinities also decrease calcification rates and slow productivity in CCA species (King and Schramm, 1982). Schoenrock et al. (2018) saw decreased calcification rates, photosynthetic efficiency, and density in *Lithothamnion glaciale* in southwest Greenland as waters became more brackish in fjord environments. Marine invertebrates are also susceptible to freshening waters as juvenile oysters cultured under decreased salinity and pH conditions showed increased rates of mortality, but low salinities alone had the most impact (Dickinson et al., 2012).

Within the Beaufort Sea, a diverse benthic community attached to boulders and cobbles occurs in an area known as the Boulder Patch (for a detailed description, see Dunton et al., 1982; Wilce and Dunton, 2014). Three kelp species (*Laminaria solidungula*, *Saccharina latissima*, and *Alaria escuelenta*) and various red algal species (e.g., *Phycodrys fimbriata* and *Coccotylus truncatus*) are common on rock surfaces (Wilce and Dunton, 2014). CCA species, including *Leptophytum foecundum* and *L. lavae*, cover 77% of the hard substratum in some areas and are completely absent in others (Konar and Iken, 2005; A. Muth pers. obs.). Benthic surveys in the Boulder Patch show patterns of decreasing CCA coverage with proximity to inshore areas with consequent increases in fleshy red algal biomass with no CCA present at the innermost sites (Muth et al., 2020). Data from salinity, temperature, and pH sensors deployed July 2016 to July 2018 in the Boulder Patch exhibit patterns of lower salinity in association with higher pH at the inshore site vs. the offshore site (Muth et al., 2020). Sites closest to the freshwater source, the Sagavanirktok River, reach low (<5) salinity levels near the benthos during ice break-up and spring flooding (late May/early June); however, this low salinity water is alkaline and pH levels increase during these pulses (Muth et al., 2020).

The mechanisms and environmental conditions that prevent CCA persistence at the inshore locations are the subject of this paper, which seeks to specifically explain the role of salinity on CCA physiology and distribution. The unique characteristics of the high alkalinity and pH levels of the Sagavanirktok River allow for a study of the influence of salinity on CCA populations, independent of pH. In general, low salinity waters (<10) have higher pH levels (>8) than high salinity (>30) waters within the Boulder Patch. We hypothesize that seawater chemistry conditions influence CCA distributions and parameters that vary with salinity (e.g., A_T_), affect CCA physiology and net calcification. Laboratory experiments were used to focus on short-term changes to CCA physiological mechanisms in response in alterations in water chemistry. Field studies allowed for real time, long-term observations of recruitment, and persistence in natural conditions.

## Materials and Methods

### Study Site and CCA Species

**Figure 1:**
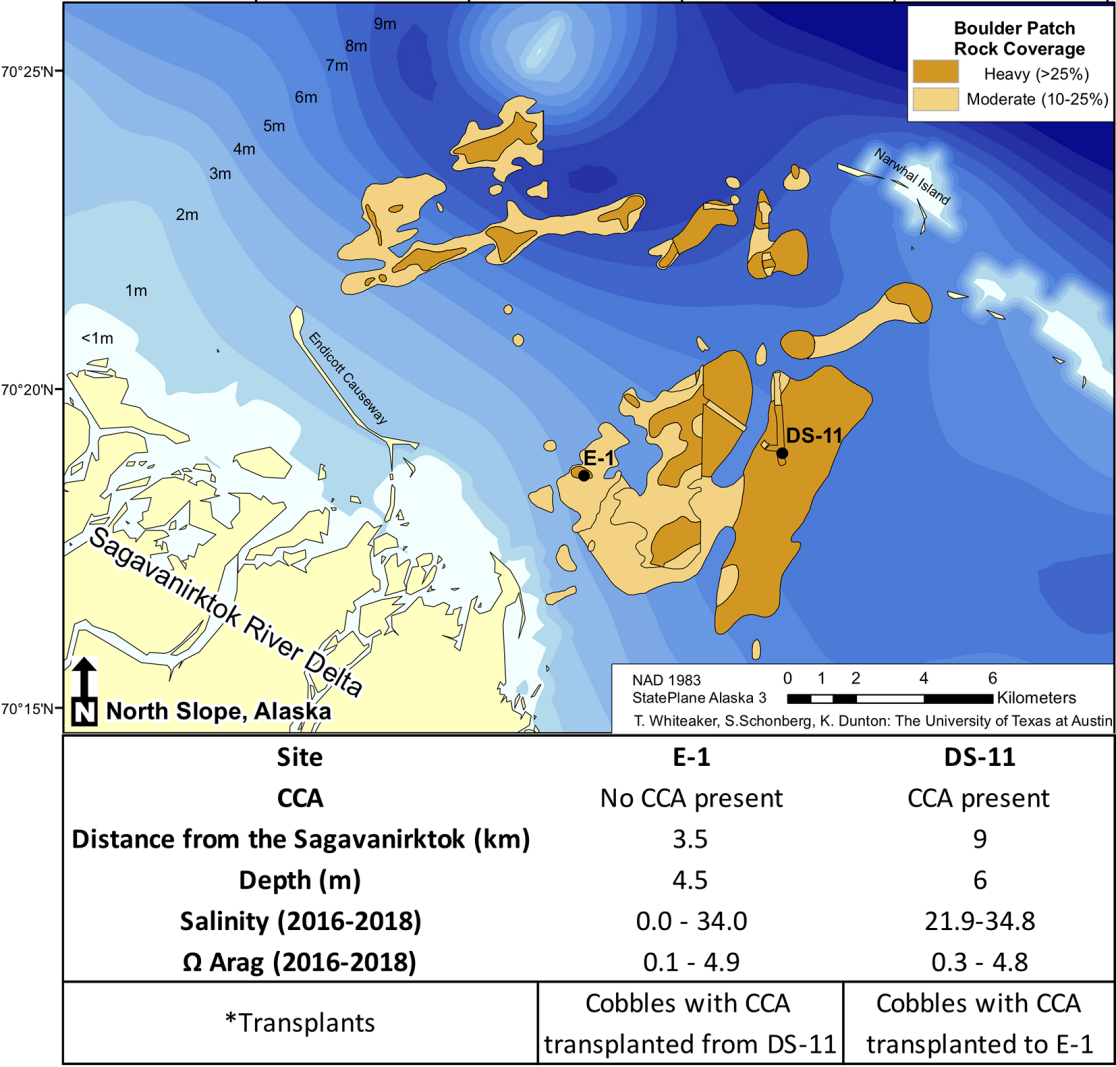
The Boulder Patch kelp bed community in Stefansson Sound, Alaska (adapted from Bonsell and Dunton, 2018 showing rock cover, site distances (km) from the Sagavanirktok River (Sag), and ranges of salinity and **Ω**_Arag_ (Muth et al., 2020).

Within the Boulder Patch, Stefansson Sound, Alaska, there are varying distributions of CCA and the most striking of these patterns is between an inshore site, located near the mouth of the Sagavanirktok River (E-1) and an offshore site (DS-11; **Figure 1**). The substrate of cobbles and boulders at the offshore site is covered by *Leptophytum foecundum* (Wilce and Dunton, 2014), while CCA is absent at the inshore site. Salinity measurements throughout the study area from past years have shown that salinity can drop to 15–20 at both sites, but can reach as low as 0 at the inshore site (Muth et al., 2020; **Figure 1**). Total alkalinity (A_T_) is often lower in fresh and brackish waters, which reduces pH buffering capacities and results in lower pH values, leading to dissolution of the calcium carbonate skeletons of calcifying organisms. However, continuous pH_T_ for the Boulder Patch show consistently higher pH values at the inshore site, where CCA are absent (Muth et al., 2020). Continuous pH values have not been available for the nearshore Arctic because of logistical constraints of deployment under the ice and instrument longevity. A_T_ values did decrease with lower salinity measurements as expected, and manipulative laboratory experiments were conducted to observe if changes in salinity and A_T_, with a constant pH, could drive dissolution and/or lower photosynthetic efficiency in *L. foecundum*.

### Manipulative Laboratory Experiments

#### Culture Conditions

Cobbles were collected in July 2016 at DS-11 (**Figure 1**) and were wrapped in damp paper towels and kept in separate plastic bags to prevent any damage to the coralline crust during transport. Corallines were then packed in a cooler within layers of Techni-Ice packs and shipped overnight to the University of Texas Marine Science Institute (UTMSI) and kept in a 0°C chamber until experiments began 15 February 2017. *Leptophytum foecundum* cobbles were cultured at undetectable light levels to replicate ambient conditions in the Arctic, at three salinities (10, 20, and 30) at 0°C. A no-specimen 30-salinity control treatment was also monitored to determine the effect of the medium on water chemistry, since we used Gulf of Mexico (GOM) water for the culture medium. Salinity treatments were chosen to represent three salinity regimes that these corallines experience throughout the year: 30 (Beaufort Sea waters predominate under stable open water conditions under the ice), 20 (mixing of offshore marine and near coastal waters), and 10 (brackish waters replace bottom saline marine waters during flooding events).

Samples (four cobbles in each tank, one tank per salinity treatment) were placed in salinity treatments (1 L of medium) for 5 weeks, after which all samples recovered at 30 (control treatment) for 5 weeks. To replicate field conditions, samples placed in low salinity (20 and 10 treatments) were placed in the control after 5 weeks for ecological relevance and to observe recovery. Growth media was created using GOM offshore water (low nutrient concentrations). Distilled water was added to achieve the salinity treatments. By diluting seawater to create salinity treatments, the ion ratios are kept constant and this method most closely mimics natural conditions as freshwater and seawater mix (Kirst, 1989). All treatments were supplemented with Provasoli’s Enriched Seawater, ensuring sufficient supply of macro- and micro-nutrients. Media was replaced weekly and water quality parameters (pH_NBS_, temperature, salinity) were recorded before and after each water change using a data sonde (YSI 6920V2-2). Water samples were also taken before and after each water change for titratable alkalinity measurements (values were log transformed to meet ANOVA assumptions of normality and equal variances).

#### Photosynthetic Efficiency

Samples were monitored weekly for photosynthetic efficiency, dark-adapted yield values (F_v_/F_m_) using a pulse amplitude modulation (PAM) fluorometer (Walz, diving-PAM). Initial baseline measurements were taken before cobbles were placed in salinity treatments. Three areas per cobble (n = 4 for each salinity treatment) were measured for yield values each week at the same location on the cobble and time of day (Measuring Light Intensity 7, Saturation Intensity 0.8, Saturation Pulse Width 8, Gain 7, and Damping 2).

#### Calcium Carbonate Calcification

Calcium carbonate calcification was estimated by measurement of A_T_ when the culture medium was first replaced (initial) and from the media after one week (final) in the experimental tanks. An automated open cell Gran titration system (ASALK2; Apollo SciTech) coupled to a thermostated water bath was used to measure A_T_ (Lonthair et al., 2017) at 0°C. A_T_ values were combined with temperature, pH, and salinity measurements to estimate aragonite saturation levels using the software program CO_2_SYS. Saturation levels equal to one are at equilibrium, greater than one favors precipitation and less than one favors dissolution.

#### Visual Pigmentation

To measure changes in visual pigmentation, cobbles were photographed before salinity treatments commenced, when placed in the recovery salinity treatments, and at the end of the experiment. Cobbles were photographed in the same position at each time point with a Nikon D7200, with each photograph containing a ruler for scale. Photographs were then analyzed using ImageJ to estimate pigmented CCA area at each time point for comparison among treatments.

### *In Situ* Field Experiments

#### Adults

To observe natural effects of abiotic factors and spatial changes in carbonate chemistry on *L. foecundum in situ*, we performed reciprocal transplants between the inshore (E-1) and offshore site (DS-11). Both sites differ considerably with respect to their hydrographic characteristics (Muth et al., 2020) with the inshore site (E-1) characterized by periods of extremely low salinities in late spring and early summer (0–20), while salinities at the offshore site (DS-11) rarely fall below 22, with values to 35 (**Figure 1**). Cobbles from offshore (n = 6) with CCA present were photographed and transferred to the inshore site and remained on the seabed from July 2016 to July 2017. Cobbles were retrieved and kept in covered buckets in ambient seawater during transit to the laboratory on Endicott Island, Alaska. In addition to the transplanted cobbles, control cobbles from each site were also collected for quantum yield value comparison in July 2017. In the laboratory, all cobbles with CCA present were measured for dark-adapted yield using PAM fluorometry (same methods as above).

#### Recruits

Fibercement tiles (10 × 10 cm) were retrieved from DS-11 and E-1 (**Figure 1**) following a 12-month deployment (July 2016 deployment) on the seabed. Tiles were attached to weighted PVC pipes with cable ties, ~3 cm from the seafloor to avoid burial by sediments. Tiles/samples were wrapped in damp paper towels and kept in separate plastic bags to prevent any damage to the recruits. Tiles were then packed in a cooler within layers of Techni-Ice packs and shipped overnight to the University of Texas Marine Science Institute (UTMSI) and kept in a 0°C chamber. Density and area of CCA recruits was quantified and compared between sites. Tiles were collected in July 2017 and recruits were observed on the inshore and offshore settlement tiles (Bonsell, 2019). Using a uniform grid, density of CCA individuals was counted for 50 FOV per tile at 40×. Pictures were taken at 40×, capturing five individuals per tile and ImageJ was used to analyze the size of the recruits from both sites.

### Statistics

Manipulative laboratory experiment parameters were compared by calculating differences in A_T_ and pH in new media and week-old media, using two-way ANOVAs (salinity treatment and treatment*recovery period) and values were log transformed in order to meet test normality and equal variance assumptions. Tukey HSD tests were used for *post hoc* comparisons. F_v_/F_m_ among treatments were compared using repeated measures two-way ANOVAs (salinity and time) during salinity treatments and following the placement of all cobbles in recovery conditions. Changes in visual pigmentation were compared using two-way ANOVAs (salinity treatment and treatment/recovery period) and ANOVAs were used to compare recruit size (values were square root transformed in order to meet ANOVA assumptions of normality and equal variances and density (values were log transformed in order to meet ANOVA assumptions of normality and equal variances) between sites. All statistics were run using R Version 3.3.1.

## Results

### Culture Conditions

Salinity and temperature treatments remained consistent over the 5-week treatment period (**Table S1**). pH values decreased in all treatments over each week (start pH 8.06 ± 0.02, end pH 7.84 ± 0.02; **Table S1**), but the changes did not differ among treatments or between periods (treatment/recovery; two-way ANOVA: period F_1_ = 0.004, p = 0.949; salinity treatment F_4_ = 1.07, p = 0.388; interaction F_4_ = 0.661, p = 0.623).

### Manipulative Laboratory Experiments

#### Photosynthetic Efficiency

**Figure 2:**
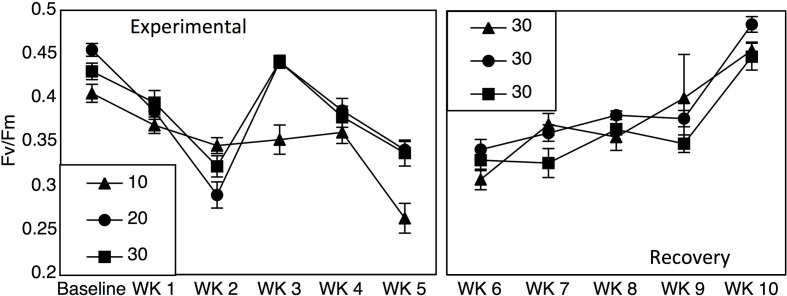
F_v_/F_m_ values (±SE) across 5 weeks of salinity treatments (10, 20, and 30) and 5 weeks in a recovery treatment of 30.

Yield values were different between the 10-salinity treatment and the 20- and 30- salinity treatments (**Figure 2**; Repeated measures two-way ANOVA: salinity F_1_ = 23.60, p < 0.001, time F_5_ = 44.33, salinity*time F_5_ = 5.47, p < 0.001). Baseline yield values were 0.40, 0.45, and 0.43 for the 10-, 20-, and 30- salinity treatments, but after 5 weeks values dropped to 0.26 in the 10-salinity treatment while the values for the 20- and 30-salinity treatment remained similar and higher (0.34 and 0.33). After cobbles were placed in a recovery salinity of 30, the previous salinity treatments did not affect yield values; however, yield values for all treatments did decrease initially and then recover over time (**Figure 2**; Repeated measures two-way ANOVA: salinity F_1_ = 0.50, p = 0.49, time F_4_ = 56.88, p < 0.001, salinity*time F_4_ = 2.14, p = 0.07).

#### Calcium Carbonate Parameters

**Figure 3:**
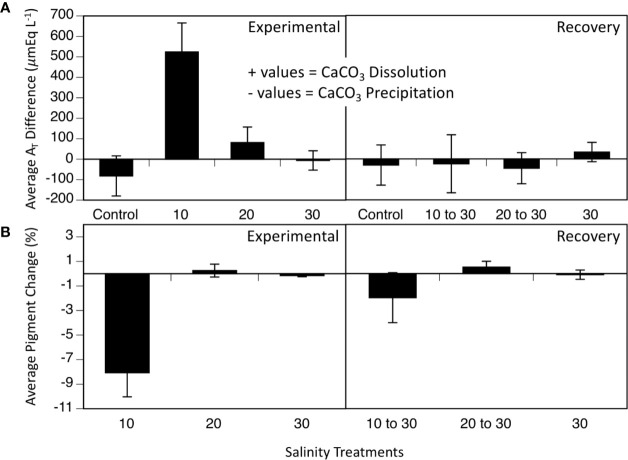
**(A)** Average A_T_ differences (n = 4) over 5 weeks at varying salinities (10, 20, 30, and control without any cobbles) and 5 weeks of recovery, when all treatments were at 30. **(B)** Average pigment loss over 5 weeks of salinity treatments (10, 20, and 30) and 5 weeks of recovery, when all treatments were at 30. Values are means ± SE.

Discrete water samples analyzed for A_T_ fluctuations each week of the experiment showed an increase in A_T_ in the 10 treatment compared to the control, 20- and 30- treatments during the 5-week period (**Figure 3**; two-way ANOVA period F_1_ = 5.39, p = 0.027; treatment F_4_ = 6.39, p < 0.001; interaction F_4_ = 4.50, p = 0.006; Tukey HSD 10–20 p = 0.03, 10–30 p = 0.004, 10-control p < 0.001; **Figure 3**, **Table 1**). Since the mesocosm is a closed system, an increase in A_T_ can only be derived from dissolution of the calcium carbonate thallus of the CCA. Media aragonite saturation levels for each treatment (**Table 2**), showed only the 10-salinity treatment was under saturation in respect to aragonite, meaning the process of dissolution was favored (Ω_Arag_ = 0.5). While the 20- and 30- treatments were at equilibrium and 1.6, respectively (precipitation/calcification favored at Ω _Arag_ > 1), dissolution did not differ between salinities in respect to A_T_ changes. Samples placed in a recovery 30 showed no significant differences in A_T_ (Tukey HSD p > 0.05).

**Table 1:** A_T_ (*μ*mEq L^−1^) values of the culture medium initially and after each week during the experimental period and recovery period (see **Figure 3**).

	Experimental Period		Recovery Period	
	**Start Average**	**Week-Old Average**	**Start Average**	**Week-Old Average**
**Control**	2512.82 (119.11)	2431.00 (117.11)	2771.72 (54.38)	2741.62 (50.55)
**10**	1208.04 (114.41)	1732.16 (63.10)	2719.36 (69.77)	2695.56 (47.42)
**20**	1870.68 (95.29)	1951.92 (71.55)	2734.06 (68.25)	2688.58 (55.60)
**30**	2550.00 (114.15)	2543.76 (77.73)	2691.54 (84.90)	2724.98 (29.49)

Values are means (± SE), n = 4.

**Table 2:** Carbonate chemistry parameters for salinity treatments used in laboratory experiments.

Salinity Treatment	HCO_3_(mmol kgSW^−1^)	CO_3_(mmol kgSW^−1^)	CO_2_(mmol kgSW^−1^)	Ω_Arag_
10	1131.1 (107.2)	32.4 (9.3)	17.2 (3.1)	0.5 (0.1)
20	1711.8 (100.1)	64.9 (13.0)	21.7 (3.6)	1.0 (0.2)
30	2293.0 (65.9)	105.4 (2.4)	25.9 (1.1)	1.6 (0.1)

Values are means (± SE).

#### Visual Pigmentation

Percent changes in visual pigmentation of the cobbles in the 10-salinity treatment lost significantly more pigmented area (8.06 % lost) during the treatment period (**Figure 3**; two-way ANOVA: period F_1_ = 4.4, p = 0.046; salinity treatment F_3,2_ = 15.56, p < 0.001, interaction F_4,5_ = 5.92, p=0.024) while the 20- and 30-treatment cobbles remained unaffected and lost little to no pigmentation throughout the experiment (0.25 % gain and 0.15% loss, respectively; **Figure 3**). Once placed in the recovery treatment of 30 salinity, loss of pigmented areas slowed in the 10 treatment (1.96 % loss; **Figure 3**, bottom panel, recovery section). As seen in all parameters measured (Fv/Fm, A_T_ changes, pigmentation changes), *L. foecundum* was affected when immersed in stressful conditions, but was able to recover when returned to the salinity of 30 medium.

### Field Experiments

#### Adults

##### Photosynthetic Efficiency and Pigmentation

**Figure 4:**
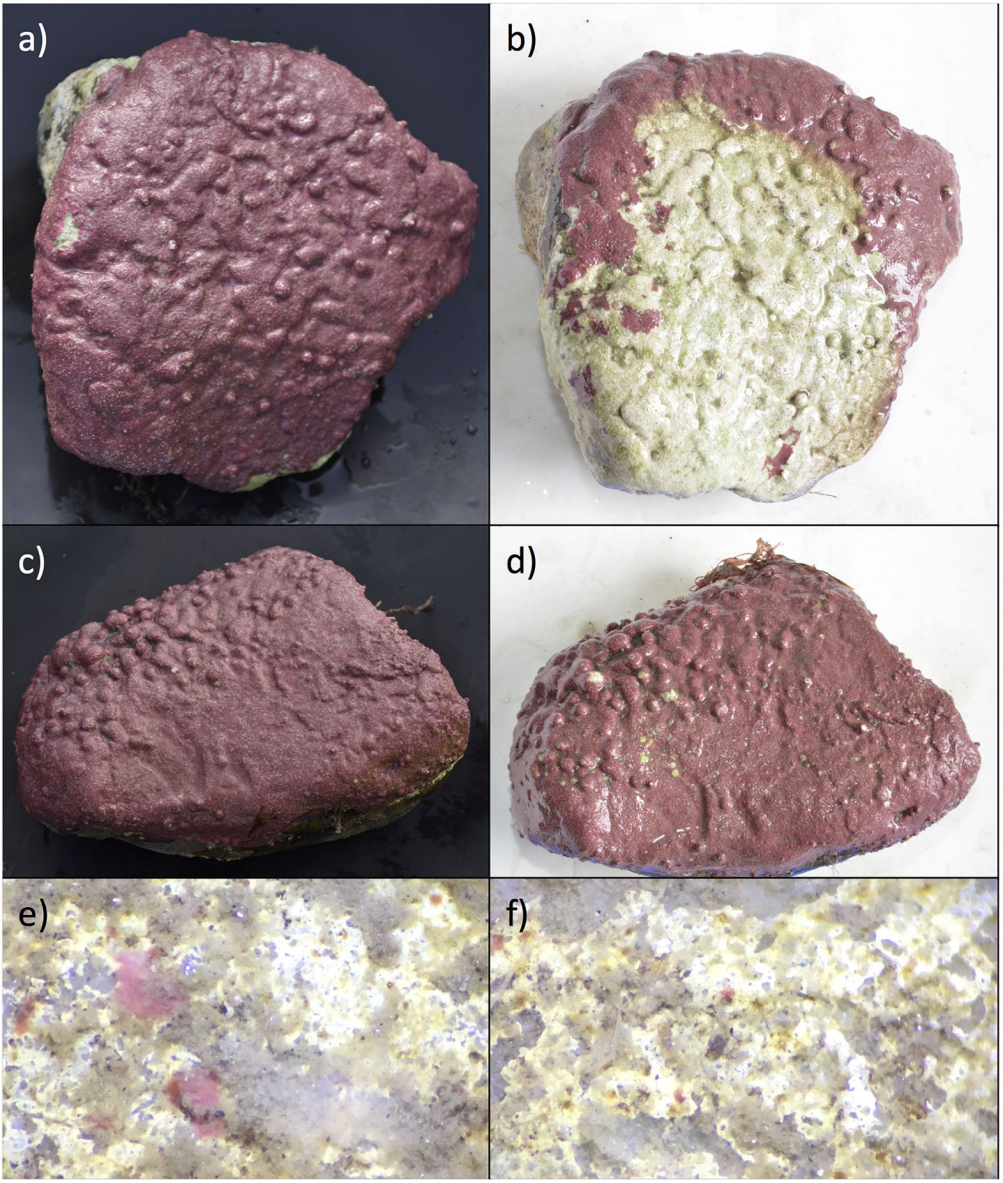
Examples of field experiments, transplanted cobbles and recruits from each site. **(A)** cobble photographed in 2016 pre-transplant **(B)** same cobble as **(A)**, photographed in 2017 following a 12-month transplant to inshore site E-1, showing high pigment loss **(C)** cobble photographed in 2016 pre-transplant **(D)** same cobble as **(C)**, photographed in 2017 following a 12-month transplant to inshore site E-1, showing low pigment loss **(E)** one year recruits at the offshore site at 400× **(F)** one year recruits at the inshore site at 400×.

Field transplants with adult CCA showed high amounts of variability. Yield (F_v_/F_m_) values were not significantly different between pigmented areas of the control and transplanted cobbles (transplanted 0.45 ± 0.01, control 0.43 ± 0.01, ANOVA F_1,34_ = 2.79, p = 0.103). Non-pigmented or dead areas (found only on the transplanted cobbles) had yield values as high as pigmented areas (0.40 ± 0.04) which we attributed to endolithic algal species living within the calcium carbonate structure. Green tinted areas had higher yield values than the control cobbles (0.53 ± 0.02). Transplanted cobbles from the offshore to the inshore site lost pigmentation (**Figures 4A–D**). However, the percent lost (23.03% ± 15.0%) was highly variable among individuals.

#### Recruits

##### Area and Density

Recruit area and density were compared between the offshore and near-shore site. Recruits were present at both sites but were significantly larger at the offshore site (**Figures 4E, F**; offshore 12.89 ± 1.91 mm^2^, inshore 1.35 ± 0.209 mm^2^; ANOVA F_1,78_ = 64.91, p < 0.001). Densities of CCA recruits were significantly higher offshore (density 1.16 ± 0.40 per cm^2^, inshore 9.02 ± 3.76 per cm^2^; ANOVA F_1,14_ = 7.02, p = 0.01).

## Discussion

Arctic coralline algae survive in an environment of high seasonal variability and extreme salinity changes (**Figures 1** and **S1**; Muth et al., 2020). Within the Boulder Patch in Stefansson Sound, CCA distributions vary from dominant benthic space holders to complete absence. The discharge of the Sagavanirktok River waters into Stefansson Sound drives changes in seawater chemistry that affect CCA and their ability to persist. Other environmental factors, such as temperature and light, vary between the two sites studied and could also influence CCA distributions. For this study, we focused on salinity to ascertain if freshwater input can influence CCA physiology. Laboratory experiments with *L. foecundum* demonstrated their sensitivity to low salinity. Reciprocal transplants between offshore station DS-11 and inshore station E-1 revealed a loss of CCA cover at E-1. These results corroborate field observations of the absence of CCA in areas in close proximity to the mouth of the Sagavanirktok River.

### Effects of Water Chemistry on *Leptophytum foecundum*

Results from mesocosm laboratory experiments illustrated that *Leptophytum foecundum*, the dominant CCA species in the Boulder Patch (Wilce and Dunton, 2014), was able to tolerate salinities to 20 without any measured physiological impacts (**Figures 2** and **3**). However, at a salinity of 10, CCA experienced reduced photosynthetic efficiency, decreased visual pigments and increased calcium carbonate dissolution (**Figures 2** and **3**). Interestingly, all parameters (F_v_/F_m_, pigmentation, and A_T_ changes) recovered rapidly once specimens were placed in a recovery salinity of 30. These results highlight that *L. foecundum* is likely very resilient to lower salinities and associated changes in carbonate chemistry. Similar resiliency was seen in other sub-Arctic coralline species, and although F_v_/F_m_ decreased at low salinities <15, the corallines did not die and were able to recover (Wilson et al., 2004). As noted by Santelices and Marquet (1998), we expect that such acclimation is related to periods of stress that the algae experience naturally on an annual basis.

**Figure 5:**
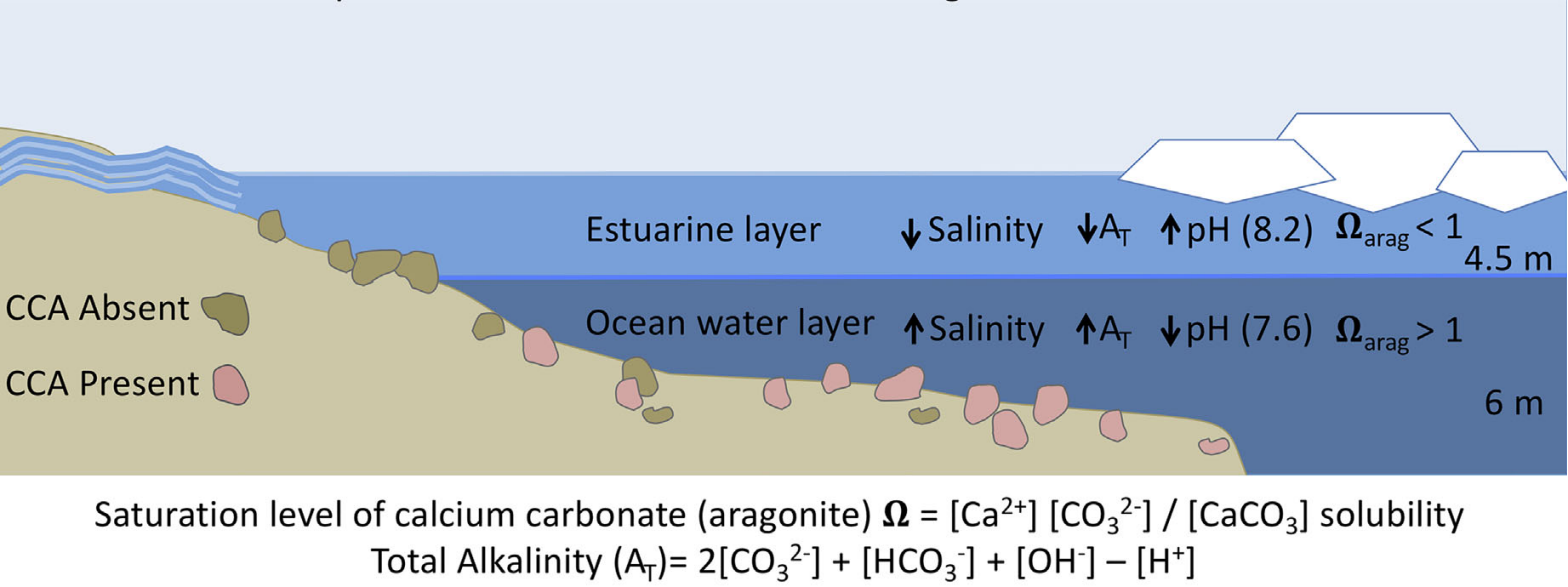
Conditions during ice break-up in the nearshore inner shelf region of the Beaufort Sea. Freshwater input from rivers peaks while remnant sea-ice reduces wave action and mixing, creating a stratified water column with a less dense brackish or estuarine water layer over cold, dense ocean water. Benthic regions within the freshwater layer experience low salinity and alkalinity conditions, driving down the aragonite saturation state, causing dissolution of the CCA populations. Lower salinity water (5–15) is defined by decreased A_T_ values (1900 *μ*mEq L^−1^) than ocean water (>30 and 2,400 *μ*mEq L^−1^). Lower A_T_ values decrease the numerator in the **Ω** equation, depressing **Ω**_Arag_ levels. This process is independent of pH, as river water pH is higher than ocean water in many near-shore Arctic systems. As break-up continues, wind driven mixing occurs, creating a homogenous water column for the summer and ice-covered seasons.

The significant (p < 0.05) increase in A_T_ in the 10 salinity treatment (**Figure 3** and **Table 1**) was driven by the 0.5 **Ω**_Arag_ level, chemically favoring net dissolution of calcium carbonate (aragonite). Dissolution of calcium carbonate is the main process that would affect A_T_ in closed mesocosm systems, especially in our culture conditions of cold temperatures and 24-h dark periods. Photosynthesis and respiration exchange neutrally charged compounds (CO_2_ and O_2_), which do not alter A_T_ (see **Figure 5** for A_T_ equation).

We kept pH levels constant among salinity treatments, and although decreases were seen in all treatments, including the control, throughout the week these did not differ significantly (p = 0.38) among treatments or between stress and recovery periods (p = 0.94). Because pH levels were similar throughout all salinity levels, results of these experiments enable us to disentangle pH and salinity. We attribute the observed physiological effects to changes in salinity and associated parameters (e.g., A_T_), but not to pH. These results are important and ecologically relevant in systems like the nearshore Arctic, where the higher alkalinity of river run-off drives pH upward to values greater than eight when compared to other freshwater sources and ice melt (Cooper et al., 2008; Semiletov et al., 2016). Although some river run-off has higher A_T_ than other freshwater sources, alkalinity levels are still lower than ocean water values (**Table 1**; Yamamoto-kawai and Tanaka, 2005; Tynan et al., 2016; Muth et al., 2020) and the increased pH of these waters does not ameliorate lower A_T_ with regard to **Ω**_Arag_ levels and calcium carbonate dissolution. These low salinity and alkalinity waters initially overlay dense ocean water, creating a stratified environment (**Figure 5**) that eventually mixes. Heterotrophic processes during the dark ice-covered period drive down pH, causing the ocean water to have lower pH values than freshwater run-off (Muth et al., 2020). The higher pH values of Alaska North Slope river waters are not sufficient to offset the influence of low A_T_ values and the resultant **Ω**_Arag_ levels. Lower pH typical of Eurasian rivers (Cooper et al., 2008) and ice melt (Yamamoto-kawai and Tanaka, 2005) would decrease **Ω**_Arag_ even more, exacerbating corrosive conditions for calcium carbonate secreting organisms.

Cobbles transplanted in the field were extremely variable in their response (pigmentation and photosynthetic efficiency) to environment change (**Figures 4A–D**). During the period of transplant (July 2016 to July 2017), salinity levels at the inshore site did not fall below 20, while the following year (2017–2018), salinity at the inshore site was near zero (**Figure 1**). These annual variations and periods of low salinity likely affect mature CCA assemblages and CCA recruitment at inshore sites, while extreme low salinity events may prevent CCA establishment entirely. In addition to changes in salinity, other factors such as sedimentation could also influence CCA distributions (Wilson et al., 2004).

The recruitment of CCA observed onto settlement tiles at the inshore site shows that the absence of CCA at the inshore site is not related to propagule dispersal limitations (Muth et al., 2020). Recruit area and density were significantly (p < 0.05) greater at the offshore site compared to the inshore site (**Figures 4E, F**). CCA propagules were seen to reach the inshore site, but their size and density were reduced when compared to the offshore site. We hypothesize that persistence of adult communities is prevented by water chemistry changes associated with low salinity pulses at the inshore site. Other CCA species (*Lithothamnion glaciale*) have shown tolerance to low salinity conditions, but as in our mesocosm studies, these studies were conducted on mature individuals (Burdett et al., 2015). However, further studies are needed to quantify and observe other factors such as grazing (Wai and Williams, 2006) and sedimentation (Wilson et al., 2004).

During the years of this study (2016–2018), **Ω**_Arag_ saturation levels were found to remain around one for much of the year (Muth et al., 2020), even at the offshore site where CCA cover the benthos. Gangstø et al. (2008) predicted that Arctic waters could reach **Ω** aragonite levels of one by 2,100, but our work has already documented these levels in Arctic near-shore environments. CCA species in the Boulder Patch survive at **Ω**_Arag_ levels near one, but during the period of spring break-up (~4–5 weeks) salinity levels drastically drop (**Figure S1**) and this, in turn, has effects on **Ω**_Arag_ levels (**Figure 1**) and these events likely drive CCA distributional patterns in Stefansson Sound.

### Ecological Implications

Predicted increases in freshwater input and the overall susceptibility of the Arctic to OA threaten to drastically change the carbonate chemistry of nearshore systems and their biological assemblages (Kelley and Lunden, 2017). This study focuses on CCA physiology and the resultant distributions, but distributions and population dynamics also affect entire communities through species interactions (Kroeker et al., 2012).

Corallines are known to grow laterally rather than vertically when colonizing space, allowing them to cover the benthos (Airoldi, 2000). However, the roles which corallines play in algal and sessile invertebrate succession vary drastically across communities. Studies have shown that corallines enhance biodiversity (Asnaghi et al., 2015) while other work has documented a complete drop in algal recruitment once coralline secure dominance (Bulleri et al., 2002). Johnson and Mann (1986) found *Phymatolithon* in Nova Scotia to suppress the recruitment of turf algal species. A similar pattern has been observed in the Stefansson Sound Boulder Patch but some algal species possess the ability to recruit to CCA (*Laminaria solidungula* and *Rhodomela confervoides*, pers. obs,). The mechanism that allows for this recruitment is unknown, but slower growing crusts (e.g., *Lithothamnion phymatodeum*; Dethier and Steneck, 2001) have shown resilience to turf overgrowth and shading. In Stefansson Sound, CCA appears to inhibit fleshy red algal occurrence but facilitates recruitment of the Arctic endemic kelp, *L. solidungula*. *Laminaria solidungula* is an ecologically important foundation species in High Arctic kelp communities that are known to support rich and diverse food webs (Dunton and Schell, 1987). Such kelp ecosystems have routinely been associated with more diverse assemblages and higher density of fishes (Bodkin, 1988; Siddon et al., 2008). Lower salinity waters from freshwater inflows and further changes to carbonate chemistry through ocean acidification not only affect the persistence of CCA, but also the community assemblages associated with these species.

The susceptibility of CCA to variations in carbonate chemistry (reviewed in Nelson, 2009) makes these species ideal sentinels as bioindicators of change in ocean chemistry in near-shore environments. Coral species in tropical zones have been used as ocean acidification bioindicators (Fabricius et al., 2012) while sea-level uplift has been estimated using CCA presence (Ortlieb et al., 1996). In the Arctic, freshwater inputs are expected to rise (Peterson et al., 2006) and few studies have considered the effects of decreased salinity in exacerbating the effects of anthropogenic OA (Schoenrock et al., 2018).

Seasonal, low salinity pulses, as seen in the Boulder Patch (Muth et al., 2020) affect CCA recruitment and growth. As freshwater input into the Arctic Ocean increases due to higher air temperatures causing an increase in snow and melt, areas devoid of CCA could increase, causing changes in epilithic communities. CCA are conspicuous benthic species, and their presence and absence could serve as an effective tool for assessing water quality changes in the nearshore Arctic that will not only affect CCA, but also other marine calcifying organisms. Increased freshwater input is not unique to the Arctic and this work highlights the importance of salinity in CCA physiology. More exploration into the functional role of CCA species is needed to fully understand ecological consequences should CCA densities decrease or disappear under future ocean conditions.

## Data Availability Statement

The datasets generated for this study are available on request to the corresponding author.

## Author Contributions

AFM designed and completed the experiment, analyzed the data, and wrote the manuscript. AE contributed to experimental design, analysis, and manuscript revisions. KHD contributed to experimental design, analysis, and manuscript revisions.

## Funding

Bureau of Ocean Energy Management M12AS00001 funded KHD and AFM for field work and travel. Environmental Protection Agency STAR fellowship funded AFM for experiments, analysis and writing.

## Conflict of Interest

The authors declare that the research was conducted in the absence of any commercial or financial relationships that could be construed as a potential conflict of interest.

## Supplementary Material

Table S1 Weekly start and end measurements of media salinity, pH and A_T_ for the experimental studies in culture.
