# Supplementary figures and images for: Physiological Responses of an Arctic Crustose Coralline Alga (Leptophytum foecundum) to Variations in Salinity
Source: Front Plant Sci. 2020 Aug 19;11:1272. doi: 10.3389/fpls.2020.01272 (PMC7466568; doi:10.3389/fpls.2020.01272)

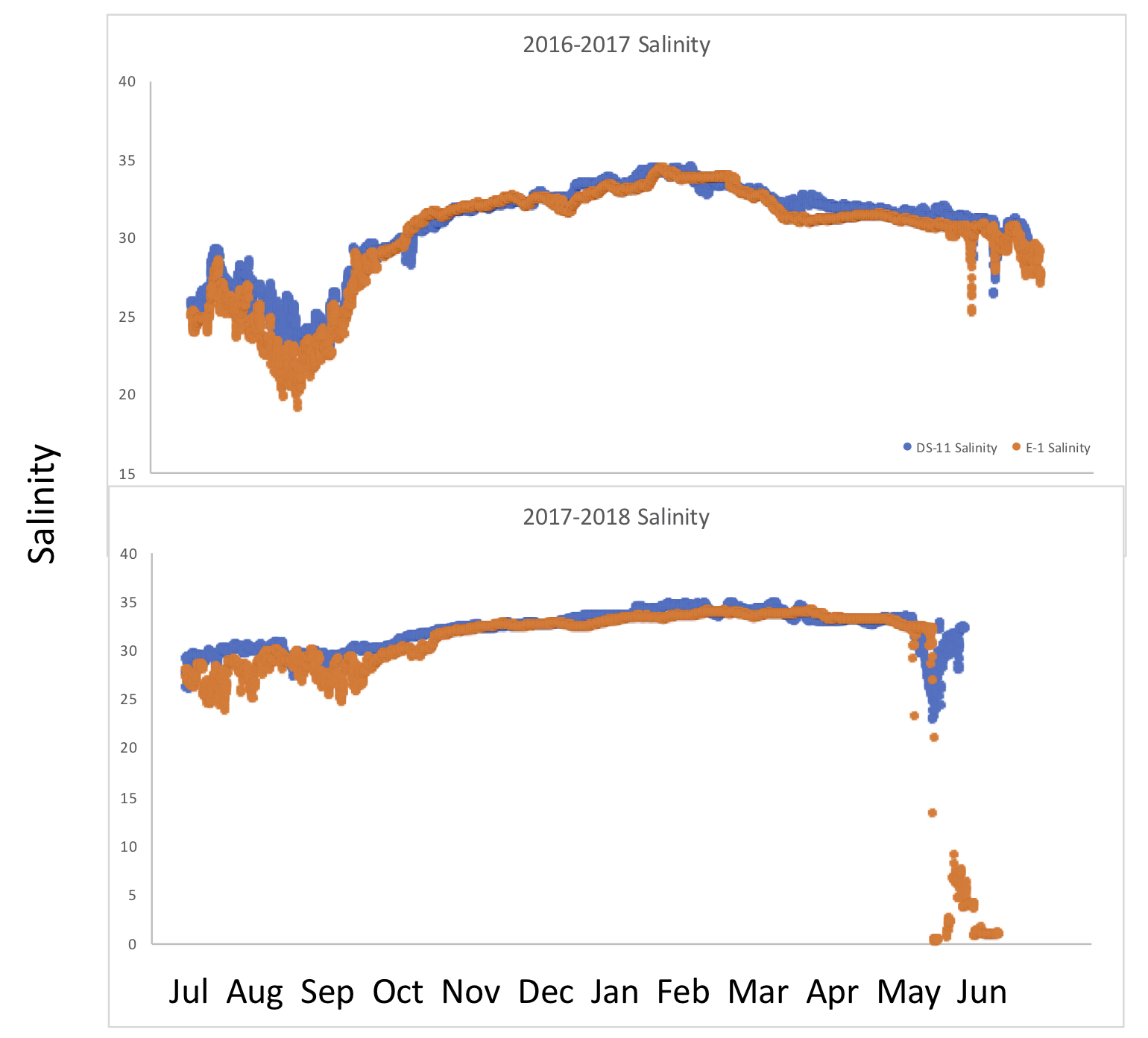

Supplement: Supplementary file 1 [file Image_1.jpeg]
