# Supplementary material for: Physiological Responses of an Arctic Crustose Coralline Alga (Leptophytum foecundum) to Variations in Salinity
Source: Front Plant Sci. 2020 Aug 19;11:1272. doi: 10.3389/fpls.2020.01272 (PMC7466568; doi:10.3389/fpls.2020.01272)
Supplement: Table S1 — Weekly start and end measurements of media salinity, pH and AT for the experimental studies in culture. [file Table_1.pdf]

	Treatment										Recovery										Averages			
	15-Feb	22-Feb	22-Feb	1-Mar	1-Mar	8-Mar	8-Mar	15-Mar	15-Mar	22-Mar	22-Mar	29-Mar	29-Mar	5-Apr	5-Apr	12-Apr	12-Apr	19-Apr	19-Apr	26-Apr				
	w1 - start	w1 - end	w2 - start	w2 - end	w3 - start	w3 - end	w4 - start	w4 - end	w5 - start	w5 - end	w6 - start	w6 - end	w7 - start	w7 - end	w8 - start	w8 - end	w9 - start	w9 - end	w10 - start	w10 - end	Treatment		Recovery	
**Salinity**																					Avg - start	Avg - end	Avg - start	Avg - end
control	30.1	30	30.03	30.23	31.48	31.36	31.61	31.45	31.52	31.73	31.82	_	31.7	31.67	31.5	31.3	31.1	28.7	29.4	29.8	30.948	30.954	31.104	30.355
10	10.21	10.6	10.06	10.38	10.3	10.81	10.25	10.43	10.32	10.4	31.69	31.39	31.95	32.34	32.24	31.01	31.1	30.96	29.8	29.75	10.228	10.524	31.356	31.33
20	21.01	20.8	20.98	21.08	20.02	21.05	21.13	21.02	20.78	20.98	31.52	31.48	30.64	31.97	32.51	30.4	31.12	31	30.7	30.95	20.784	20.986	31.298	31.16
30	30.8	31	31	30.98	31.86	31.87	31.62	31.82	32.06	31.98	31.37	31.47	31.88	31.88	31.87	31.6	31.08	31.02	30.82	30.96	31.436	31.458	31.404	31.386
																					Treatment		Recovery	
**AT**																					Avg - start	Avg - end	Avg - start	Avg - end
control	2474.3	2459.8	2156	2144.2	2382.7	2394.8	2776.1	2307.3	2775	2848.9	2639.1	2821.2	2653.5	2649.3	2788.9	2773.6	2888.3	2599.6	2888.8	2864.4	2642.27	2586.31	2771.72	2741.62
10	1296.6	1565.1	972.6	1842.2	1525.6	1868.2	1326.6	1597.8	918.8	1787.5	2523.6	2569.8	2607.8	2619.2	2898.2	2796.4	2840	2809.6	2727.2	2682.8	1208.04	1732.16	2719.36	2695.56
20	1817.4	1768.7	1684.1	2027	1742.7	1905.5	2227.7	2185.9	1881.5	1872.5	2538.4	2492.9	2601.2	2639	2828.7	2777.8	2872.7	2788.5	2829.3	2744.7	1870.68	1951.92	2734.06	2688.58
30	2492.7	2428.8	2228.2	2397.8	2528.6	2529.4	2559	2526.1	2941.5	2836.7	2726.9	2673.6	2366.7	2652.7	2767.6	2804.5	2864.7	2780.5	2731.8	2713.6	2550	2543.76	2691.54	2724.98
																					Treatment		Recovery	
**pH**																					Avg - start	Avg - end	Avg - start	Avg - end
control	8.3	7.93	8.03	7.8	8.03	7.97	8.06	7.74	8.03	8.07	8.01	_	8.03	7.82	8.01	7.74	8.03	7.94	8.01	7.83	8.09	7.902	8.018	7.8325
10	8.49	8.01	8.01	7.9	8.04	8.04	8.03	7.87	8.02	7.94	8.03	7.73	8.06	7.98	8.03	7.58	8.03	7.85	8.05	7.8	8.118	7.952	8.04	7.788
20	8.44	7.92	8.07	7.85	8.03	7.89	8.04	7.78	8	7.79	8.05	7.85	8.02	7.81	8.01	7.65	8.03	7.8	8.08	7.8	8.116	7.846	8.038	7.782
30	8.3	7.74	8.02	7.93	8.05	7.81	8.07	7.65	8.01	7.76	8	7.92	8.01	7.78	8.02	7.71	8.03	7.95	8.09	7.82	8.09	7.778	8.03	7.836
